# Robotic-assisted versus standard laparoscopic radical cystectomy in bladder cancer: A systematic review and meta-analysis

**DOI:** 10.3389/fonc.2022.1024739

**Published:** 2022-11-09

**Authors:** Junhao Long, Li Wang, Ni Dong, Xiaoli Bai, Siyu Chen, Shujun Sun, Huageng Liang, Yun Lin

**Affiliations:** ^1^ Department of Anesthesiology, Union Hospital, Tongji Medical College, Huazhong University of Science and Technology, Wuhan, China; ^2^ Department of Urology, Union Hospital, Tongji Medical College, Huazhong University of Science and Technology, Wuhan, China; ^3^ Department of Anesthesiology, Eye Hospital of Hebei Province, Xingtai, China

**Keywords:** bladder cancer, laparoscopy, robotics, randomized controlled trials, meta-analysis

## Abstract

**Background:**

This study aimed to evaluate the efficacy and safety of robotic-assisted radical cystectomy (RARC) versus laparoscopic radical cystectomy (LRC) in the treatment of bladder cancer.

**Methods:**

Two researchers independently searched PubMed, Embase, Cochrane, and CBM using the index words to identify the qualified studies which included randomized controlled trials (RCTs) and non-randomized controlled trials (prospective and retrospective studies), and the investigators scanned references of these articles to prevent missing articles. Differences in clinical outcomes between the two procedures were analyzed by calculating odds risk (OR) and mean difference (MD) with an associated 95% confidence interval (CI).

**Results:**

Sixteen comparative studies were included in the meta-analysis with 1467 patients in the RARC group and 897 patients in the LRC group. The results indicated that RARC could significantly decrease blood loss (*P* = 0.01; MD: -82.56, 95% CI: -145.04 to -20.08), and complications 90 days or more after surgery, regardless of whether patients were Grade ≤ II (*P* = 0.0008; OR: 0.63, 95% CI: 0.48 to 0.82) or Grade ≥ III (*P* = 0.006; OR: 0.59, 95% CI: 0.40 to 0.86), as well as overall complications (*P*: 0.01; OR = 0.52; 95% CI: 0.32 to 0.85). However, there was no statistical difference between the two groups at total operative time, intraoperative complications, transfusion rate, short-term recovery, hospital stay, complications within 30 days of surgery, and bladder cancer-related mortality.

**Conclusions:**

The meta-analysis demonstrates that RARC is a safe and effective treatment for bladder cancer, like LRC, and patients with RARC benefit from less blood loss and fewer long-term complications related to surgery, and should be considered a viable alternative to LRC. There still need high-quality, larger sample, multi-centric, long-term follow-up RCTs to confirm our conclusion.

## Introduction

Bladder cancer is the 12th most common malignancy in the world, accounting for approximately 3.0% of all new cancer diagnoses and 2.1% of all cancer deaths in 2020, according to new reports (1). Alarmingly, the prevalence of bladder cancer has risen in many countries, especially in Europe (2). Open radical cystectomy (ORC) is the gold standard for the treatment of non-metastatic muscle-invasive and uncontrolled or high-risk superficial bladder cancer, which can effectively achieve local control of the tumor and long-term disease-free survival (3, 4). However, traditional ORC often has high surgical risks, many perioperative complications and high mortality, and previous research data show that the incidence of postoperative complications after ORC is as high as 30% to 60%, even if the surgeon knows enough about pelvic anatomy and the surgical technique is continuously improved (5). Therefore, minimally invasive surgery for bladder cancer is still necessary.

In the early 1990s, Parra et al. (6) and Sánchez de Badajoz et al. (7) reported the use of LRC for muscle-invasive bladder cancer, which had less intraoperative blood loss, less postoperative pain, faster postoperative bowel function recovery, and shorter hospital stay compared with ORC (8). After a decade, Menon et al. (9) reported the use of robotic-assisted radical cystectomy (RARC) in the treatment of bladder cancer, which was later adopted by many large medical units and proved to be feasibility. Later, a large number of studies compared RARC with ORC, the gold standard for the treatment of invasive bladder cancer, and also found that RARC can achieve the same radical effects as ORC in terms of lymph node count, positive surgical margins, and survival rate; but the RARC group had less intraoperative blood loss, lower blood transfusion rate, shorter postoperative exhaust time, and lower incidence of surgery-related complications, especially for elderly patients (10–13). More recently, the Catto et al. (14) study also demonstrated that, compared with ORC, RARC offered more days alive and out of the hospital within 90 days of surgery, less thrombo-embolic complications and wound complications, and a better quality of life at 5 weeks.

Currently, LRC and RARC appear to be safe and viable alternatives to ORC as they mature. Tang K et al. and Li K et al. performed meta-analysis of LRC and ORC, RARC and ORC respectively, the results of which show that the minimally invasive endoscopic technique (LRC and RARC) has reliable perioperative safety, and can achieve the same tumor resection effects and function of reconstruction bladder as ORC, meanwhile, has lower surgery-related complications than ORC (15, 16). However, there is a lack of evidence for the multicenter, large sample of controlled studies on which RARC and LRC are more advantageous in radical surgery for bladder cancer. Thus, the meta-analysis aimed to obtain a more powerful evaluation of the use of LRC versus RARC in the treatment of bladder cancer by incorporating more recent studies.

## Materials and methods

### Search strategy

The databases of PubMed, Embase, Cochrane, and CBM were searched to determine these qualified studies comparing the efficacy of RARC versus LRC in the treatment of bladder cancer. The mesh (cystectomy, laparoscopy, and robotic surgical procedures) and their corresponding keywords used for the searches, and the search strategy are detailed in the appendix. In addition, the investigators scanned other related articles and reference materials for these articles to prevent missing articles. The literature search was done independently by two investigators and was resolved by discussing with the third investigator when the search results were inconsistent.

### Inclusion and exclusion criteria

The study was included in our meta-analysis if it was: (1) English and Chinese articles; (2) the research subjects were patients with bladder cancer and no other serious cardiopulmonary vascular diseases; (3) the study included at least two groups (RARC group and LRC group); (4) report at least one result of interest to us; (5) no time limit for publication of included articles.

The study was excluded in our meta-analysis if it was: (1) a duplicate article; (2) the data had obvious mistakes; (3) the case report, theoretical research, conference report, systematic review, meta-analysis, expert comment, or economic analysis; (4) we went through various means but still could not get the full text of this study.

The screening process of the eligible studies was completed by two reviewers independently and was resolved by discussing with the third reviewer when there was a disagreement.

### Data extraction and quality assessment

The data, extracted from all included studies, consists of two parts: basic information and main results. The basic information includes the first author’s name and publication time, country, study design, the sample size of interventions and control groups, matching/comparable variables, conversion (N), and follow-up time. The clinical outcomes excerpted were used for statistical analysis, including total operative time, blood loss, blood transfusion rate, length of hospital stay, days to oral intake and regular diet, complications, and oncologic outcomes. Two investigators independently assessed the methodological quality of randomized controlled trials (RCTs) using the Jadad scale, while the Methodological Index for Non-Randomized Studies (MINORS) tool was used to assess the methodological quality of the non-randomized controlled study (NRS) (17, 18). The Jadad scale focuses on randomization, blinded, and reported dropout, where the literature mentions the application of randomized methods and double-blind (+1) and correct methods (+2). The number of cases of withdrawal and loss of follow-up and the reasons for withdrawal were described in detail (+1) or not, and the total score > 2 were high-quality clinical trials. The MINORS tool contains a total of 12 items for the comparative studies, and each item is scored 0 to 2 points (0 = not reported; 1 = reported but insufficient information; 2 = reported and provided sufficient information), and the article was divided into low (> 17), moderate (≥ 10 and ≤ 17) and high bias risk (< 10) according to the methodological quality score (18). All of the above data extraction and quality evaluation processes were completed independently by two reviewers and disagreements between reviewers were resolved through discussion until a consensus was reached.

This meta-analysis does not require Institutional Review Board (IRB) approval.

### Statistical analysis

All statistical analyses in the meta-analysis were performed using the RevMan version 5.3 (The Cochrane Collaboration, Oxford, UK). The results of Chi-squared and *I^2^* tests were used to assess the heterogeneity and determine which analytical model (fixed-effect or random effect model) to use for data integration (19).

Assuming the chi-square test with a *P* value of ≤ 0.05 and the *I^2^* test value > 50% were defined as the presence of greater heterogeneity and a random effects model was used for data analysis, meanwhile, we performed subgroup analysis or sensitivity analysis to find possible sources of heterogeneity and eliminate heterogeneity as much as possible. Conversely, if the Chi-squared *P* value of > 0.05 and the *I^2^* test value ≤ 50%, the heterogeneity between the data was considered to be small, and the data analysis used a fixed-effect model. Continuous variables are expressed as the mean ± standard deviation (SD) and analyzed by mean difference (MD). In addition, clinical outcome measures were reported in the median and range or interquartile range in some studies. For ease of integration, mean and SD were generated by network calculators (http://www.comp.hkbu.edu.hk/~xwan/median2mean.html) based on the sample size, median, and range or interquartile range. Categorical data are presented as percentages and analyzed by odds risk (OR). Data associated with blood transfusion rate and complications and oncologic outcomes were analyzed by OR with 95%CI. MD along with 95% CI were used to analyze the data associated with total operative time, blood loss, length of hospital stay, days to oral intake, and regular diet.

## Results

### Characteristics of the included studies

**Figure 1:**
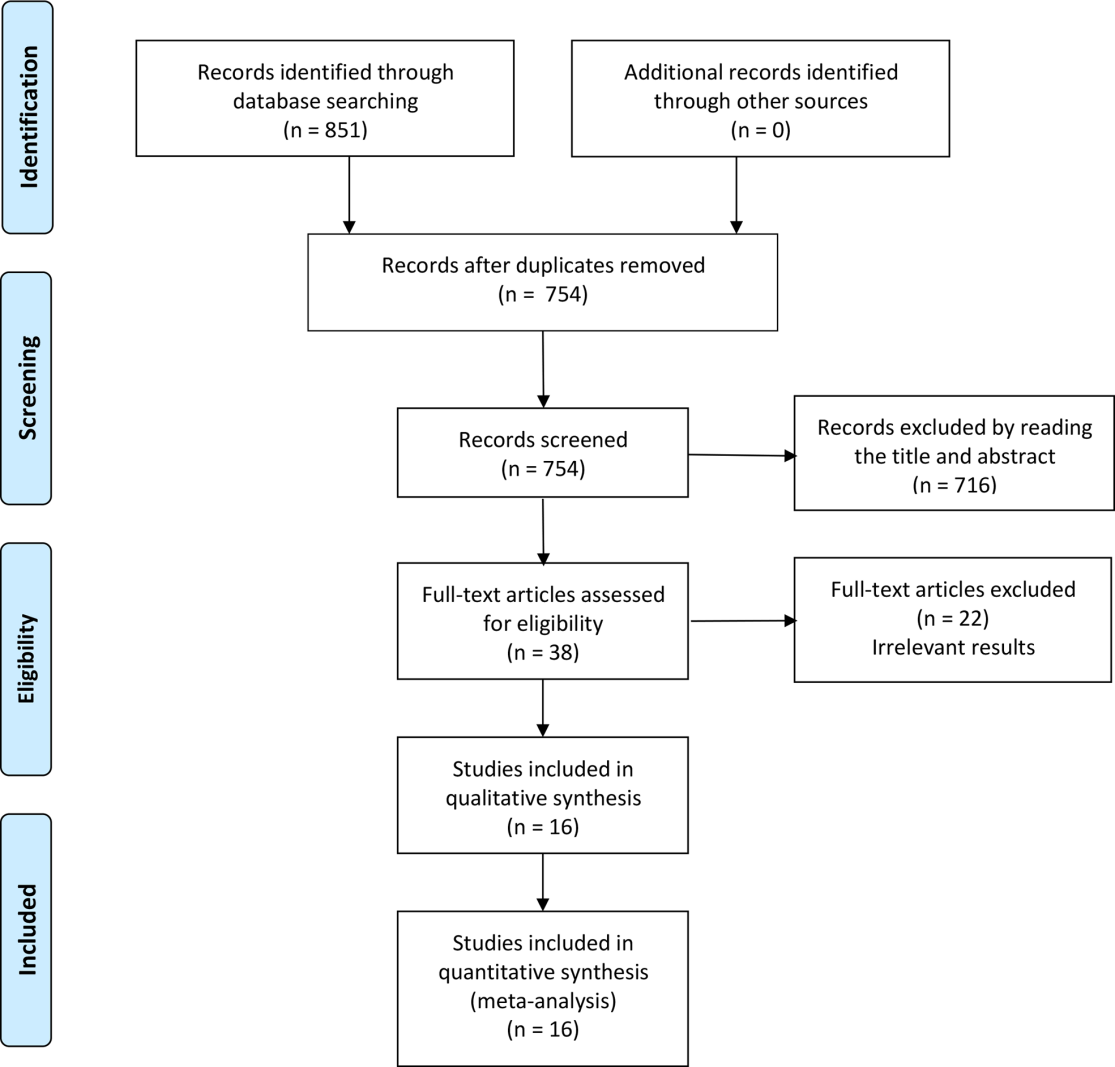
Flow diagram of the literature search and selection process.

A total of 851 articles were identified by searching, of which 97 articles are duplicates. 716 articles were excluded by reading the title or abstract of the studies, and 38 articles were left for further evaluation. After obtaining and reading the full text, 22 articles were further excluded, at last, 16 articles (four Chinese and twelve English) (20–35) were involved in the meta-analysis, which was performed with 1467 patients in the RARC group and 897 patients in the LRC group. The flow chart is presented in **Figure 1**. The basic information of the included studies is presented in **Table 1**. The risk assessment of the included studies is shown in **Table 2**. The only RCT of included studies had a Jadad scale score of 3, and the mean MINORS score is 16.69 ± 1.72, indicating that the quality of evidence from the included studies was moderate.

**Table 1:** The basic characteristics description of included studies.

Study	Country	Study design	Patients (n):RARC/LRC	Matching/comparable variables	Conversion (N): RARC/LRC	Follow-up (months)
Abraham JB 2007 (6)	USA	prospective	14/20	1,2,3,4,5,6,7	0/3	NA
Gastecka, A 2018 (15)	Poland	retrospective	52/37	1,2,3,6,7	NA	1/1
Jia GZ 2017 (16)	China	retrospective	38/61	1,2,3,4,5,6,7	0/0	NA
Khan, M. S 2012 (17)	UK	prospective	48/58	1,2,4,5,6,7	0/1	38.4/38.4
Khan, M. S 2016 (18)	UK	RCT	20/19	1,2,3,4,6,7	0/1	12/12
Kim, T. H 2016 (19)	Korea	retrospective	58/22	1,2,3,4,6,7	NA	32.0/28.8
Matsumoto K 2019 (20)	Japan	prospective	10/10	1,2,3,4,6,7	NA	NA
Teishima, J 2014 (21)	Japan	prospective	6/5	1,3,5,7	0/0	1/1
Wei XS 2016 (22)	China	retrospective	6/57	1,2,3,4,5,6,7	0/0	NA
Bai, YC 2021 (23)	China	retrospective	136/82	1,2,3,4,5	NA	33.0/33.0
Arora, A 2020 (24)	France	retrospective	188/112	1,2,3,4,5,7	5/5	NA
Su SQ 2019 (25)	China	retrospective	189/126	1,2,3,4,5,6,7	NA	34.2/34.2
Zhang SW 2019 (26)	China	retrospective	172/126	1,2,3,4,5,6	0/0	NA
Porreca A 2022 (27)	Italy	prospective	368/46	1,2,3,4,5,6,7	25/2	24/24
Jiang S 2022 (28)	China	retrospective	87/32	1,2,3,5,7	0/0	NA
Huang XM 2019 (29)	China	retrospective	75/84	1,2,3,5,6,7	NA	NA

NA, data not available; Matching/comparable variables: 1 = age, 2 = gender, 3 = BMI, 4 = ASA, 5 = Previous surgery history, 6 = Urinary diversion type, 7 = pathological stage.

**Table 2:** Risk of bias for the involved studies.

The quality of NRS was evaluated with the MINORS															
Methodological Items for non-randomized studies	Abraham JB 2007 (6)	Gastecka, A 2018 (15)	Jia GZ 2017 (16)	Khan, M. S 2012 (17)	Kim, T. H 2016 (19)	Matsumoto K 2019 (20)	Teishima, J 2014 (21)	Wei XS 2016 (22)	Bai YC 2021 (23)	Arora, A 2020 (24)	SuSQ2019 (25)	ZhangSW 2019 (26)	Porreca,A2022 (27)	Jiang S 2020 (28)	Huang XM2019 (29)
Clearly Stated Aim	2	2	2	2	2	2	2	2	2	2	2	2	2	2	2
Consecutive Patients	2	1	1	1	2	2	0	1	2	1	2	1	2	1	1
Prospective Data Collection	2	2	2	2	2	2	2	2	2	2	2	2	2	2	2
Appropriate Endpoint	2	2	2	2	2	2	2	2	2	2	2	2	2	2	2
Unbiased Endpoint Assessment	1	0	0	0	0	0	0	0	0	0	0	0	0	0	0
Appropriate Follow-Up	1	0	0	1	0	1	1	0	1	0	1	0	1	0	0
Loss to Follow-Up <5%	2	0	0	2	0	2	2	0	1	0	2	0	2	0	0
Prospective Study Size Calculation	0	0	0	0	0	0	0	0	0	0	0	0	2	0	0
An adequate control group	2	2	2	2	2	2	2	2	2	2	2	2	2	2	2
Contemporary groups	2	2	2	2	2	2	2	2	2	2	2	2	2	2	2
Baseline equivalence of groups	2	2	2	2	2	2	2	2	2	2	2	2	2	2	2
Adequate statistical analyses	2	2	2	2	2	2	2	2	2	2	2	2	2	2	2
Score	20	15	15	18	16	19	17	15	18	15	19	15	21	15	15
The quality of remaining RCTs were assessed using the Jadad scale															
Khan, M. S 2016 (18)	3														

### Total operative time

**Figure 2:**
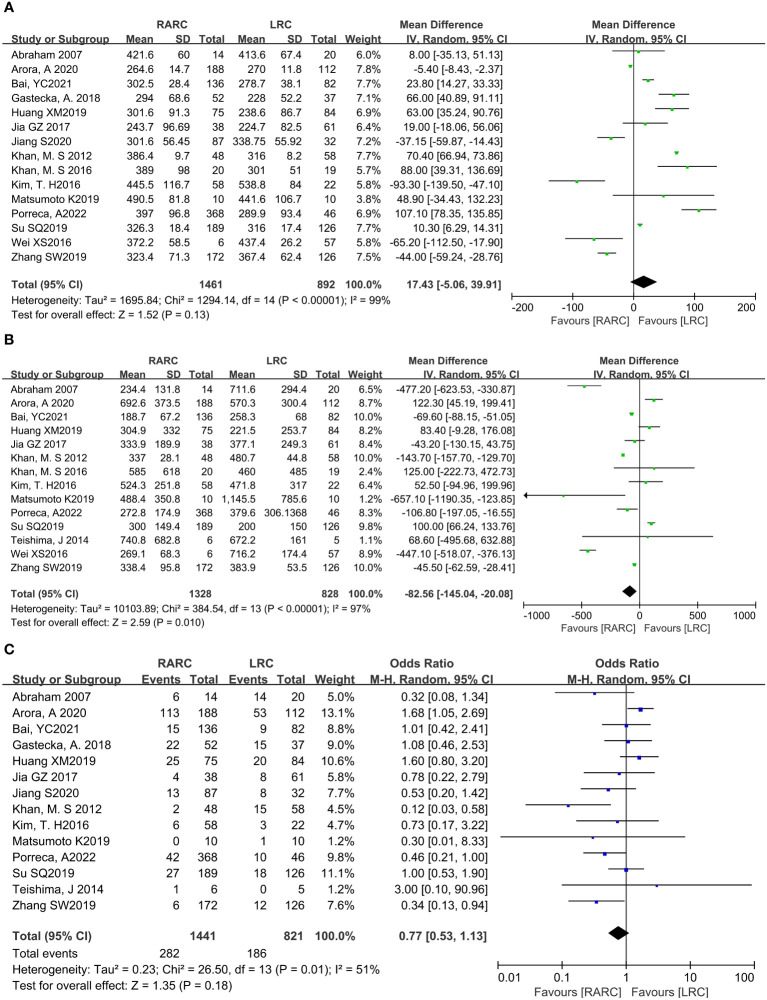
Forest plot of RARC versus LRC on **(A)** Total operative time, **(B)** Amount of blood loss, and **(C)** Transfusion rate.

Fifteen studies with 2353 patients (RARC group = 1461, LRC group = 892) reported the total operative time. Based on the Chi-squared (*P* < 0.001) and *I^2^* test (*I^2^* = 99%), we used the random effect model to analyze the total operative time. The pooled results show there was no significant difference in the total operative time between the two groups (*P* = 0.13; MD: 17.43, 95% CI: -5.06 to 39.91, **Figure 2A**).

### Amount of blood loss

Fourteen studies with 2156 patients (RARC group = 1328, LRC group = 828) reported the amount of blood loss. Based on the Chi-squared (*P* < 0.001) and *I^2^* tests (*I^2^* = 97%), we used the random effect model to analyze the amount of blood loss. Compared with LRC, the amount of blood loss during RARC was reduced at a statistically significant level (*P* = 0.01; MD: -82.56, 95% CI: -145.04 to -20.08, **Figure 2B**).

### Transfusion rate

Fourteen studies with 2262 patients (RARC group = 1441, LRC group = 821) reported the transfusion rate. Based on the Chi-squared (*P* = 0.01) and *I^2^* tests (*I^2^* = 51%), we used the random effect model to analyze the transfusion rate. There was no significant difference in the transfusion rate between the two groups (*P* = 0.18; OR: 0.77, 95% CI: 0.53 to 1.13, **Figure 2C**).

### Hospital stay

**Figure 3:**
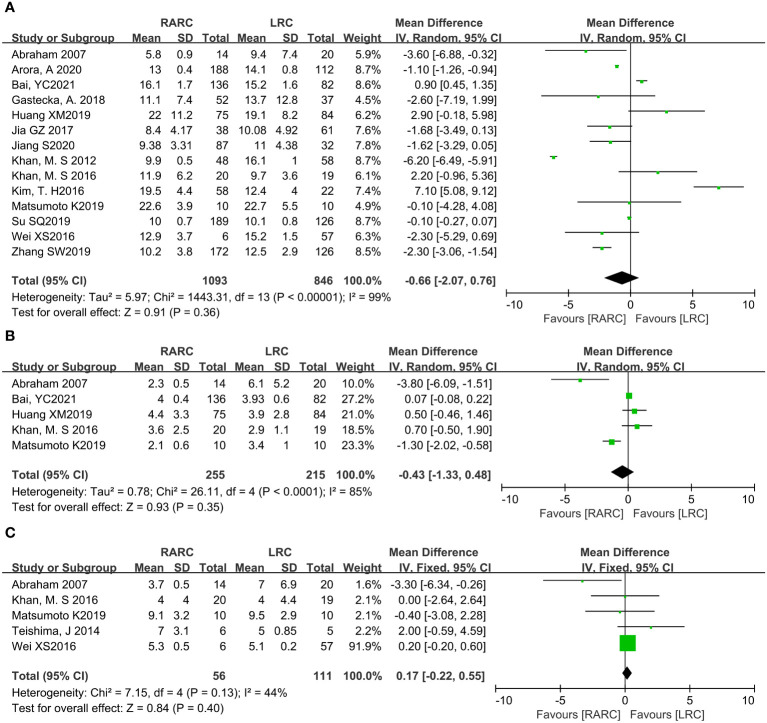
Forest plot of RARC versus LRC on **(A)** Hospital stay, **(B)** Days to oral intake, and **(C)** Days to regular diet.

Fourteen studies with 1939 patients (RARC group = 1093, LRC group = 846) reported the hospital stay. Based on the Chi-squared (*P* < 0.001) and *I^2^* tests (*I^2^* = 99%), we used the random effect model to analyze the hospital stay. Compared with LRC, the hospital stay of RARC no significant difference (*P* = 0.36; MD: -0.66, 95% CI: -2.07 to 0.76, **Figure 3A**).

### Short-term recovery

For short-term recovery, we analyzed “day to oral intake” and “day to regular diet”, where day to oral intake included five studies with 470 patients (RARC group = 255, LRC group = 215), and day to regular diet included five studies with 167 patients (RARC group = 56, LRC group = 111). The summary results showed no significant difference in the short-term recovery between the two groups, whether the day to oral intake (*P* = 0.35; MD: -0.43, 95% CI: -1.33 to 0.48) or the day to regular diet (*P* = 0.40; MD: 0.17, 95% CI: -0.22 to 0.55), as shown in **Figures 3B, C**.

### Oncologic outcomes

**Figure 4:**
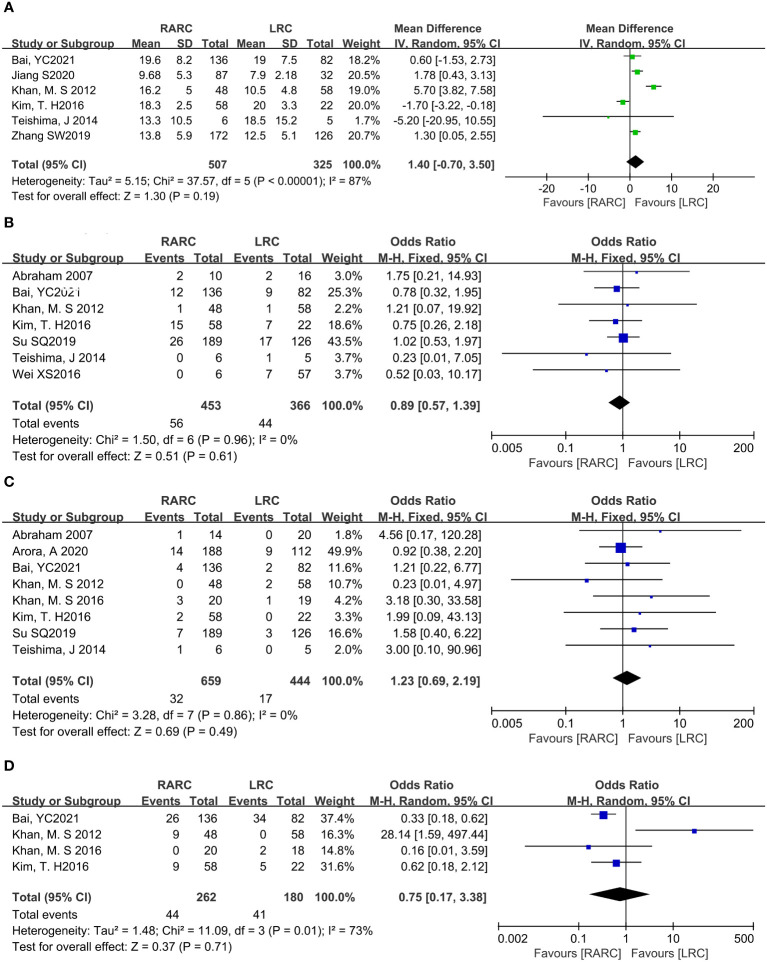
Forest plot of RARC versus LRC on the oncologic outcomes. **(A)** The mean lymph nodes, **(B)** The positive lymph nodes, **(C)** The positive surgical margins, and **(D)** Cancer-related mortality.

Mean lymph node yield and positive lymph node. Pooling data from six studies that counted lymph node yield in 832 patients (RARC group = 507, LRC group = 325) and seven studies including 819 patients (RARC group = 453, LRC group = 366) who reported positive lymph nodes, there was no significant difference between the two groups in terms of mean lymph nodes yield (*P* = 0.19; MD: 1.40, 95% CI: -0.70 to 3.50) or positive lymph node (*P* = 0.61; OR: 0.89, 95% CI: 0.57 to 1.39), as shown in **Figures 4A, B**.

Positive surgical margins. Pooling data of eight studies that reported positive surgical margins in 1103 patients (RARC group = 659, LRC group = 444) also showed that there was no significant difference between the two groups (*P* = 0.49; OR: 1.23, 95% CI: 0.69 to 2.19, **Figure 4C**).

Cancer-related mortality. At the same time, we analyzed bladder cancer-related mortality, which was reported in a total of 442 patients (RARC group = 262, LRC group = 180), and the integration showed no significant difference between the two groups (*P* = 0.71; OR: 0.75, 95% CI: 0.17 to 3.38, **Figure 4D**).

### Complications

The surgical-related complications were graded according to Clavien-Dindo (36) and were combined into two categories according to whether the complications required surgical intervention (Grade ≤ II and Grade ≥ III). According to the time of occurrence of surgery-related complications, we divided them into intraoperative complications, early complications 30 days after surgery, and long-term complications 90 days or more after surgery, and analyzed the total complication rates of the two groups.

**Figure 5:**
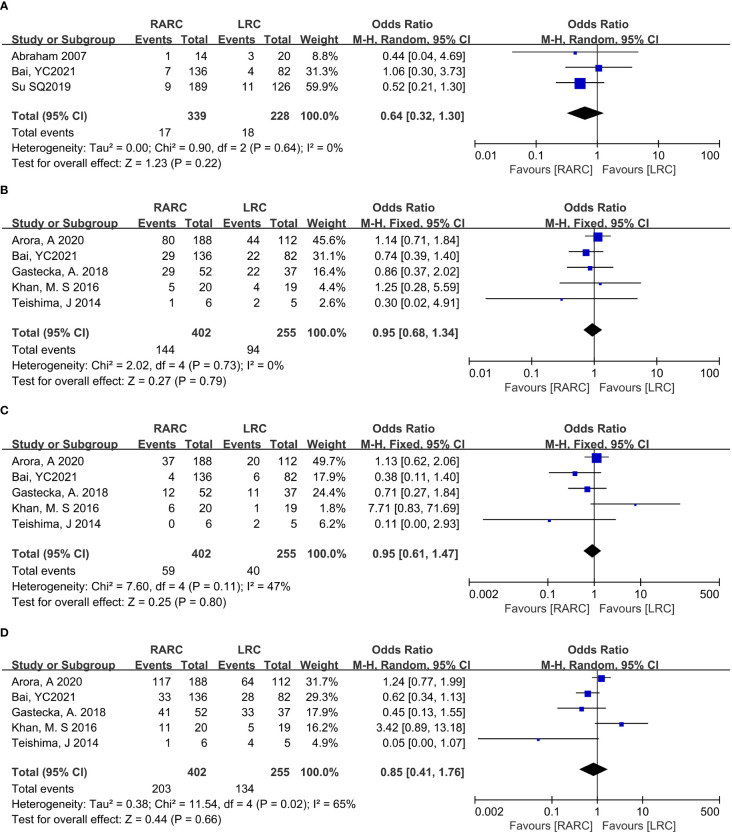
Forest plot of RARC versus LRC on **(A)** Intraoperative complication, **(B)** Postoperative complication Grade ≤ II within 30 days, **(C)** Postoperative complication Grade ≥ III within 30 days, and **(D)** Early complications within 30 days.

Pooling data of three studies including 567 patients (RARC group = 339, LRC group = 228) who reported intraoperative complications, and five studies including 657 patients (RARC group = 402, LRC group = 255) described the occurrence of surgery-related complications within 30 days of surgery. Forest plot showing that there was no significant difference on intraoperative complications (*P* = 0.22; OR: 0.64, 95% CI: 0.32 to 1.30), and 30-day postoperative complications, whether it was Grade ≤ II (*P* = 0.79; OR: 0.95, 95% CI: 0.68 to 1.34) or Grade ≥ III (*P* = 0.8; OR: 0.95, 95% CI: 0.60 to 1.47), and total early surgery-related complication rates (*P* = 0.66; OR: 0.85, 95% CI: 0.41 to 1.76) compared with LRC group, as shown in **Figure 5**.

**Figure 6:**
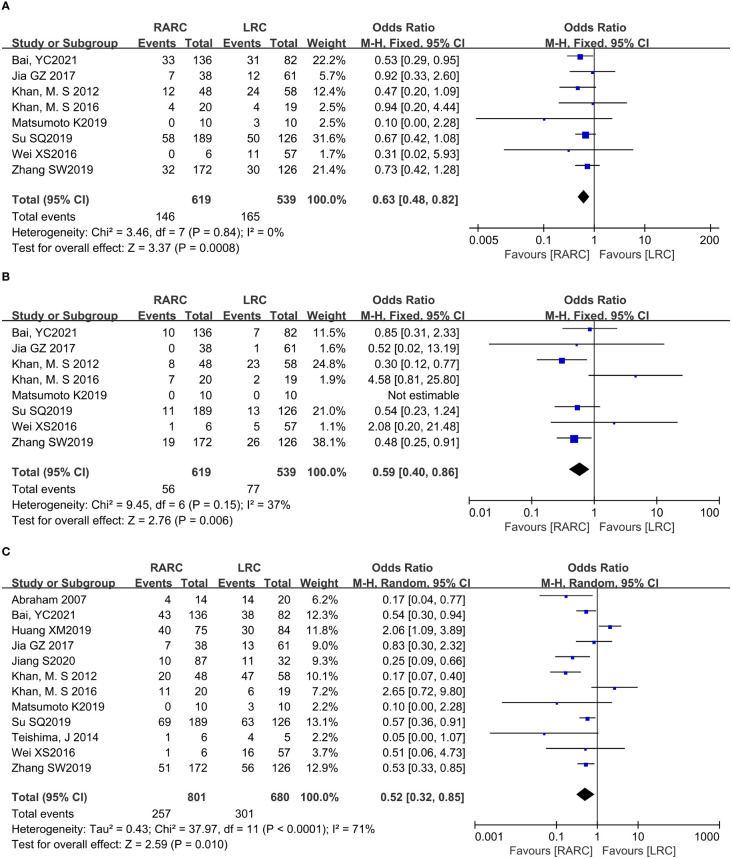
Forest plot of RARC versus LRC on **(A)** Complication Grade ≤ II postoperative ≥ 90 days, **(B)** Complication Grade ≥ III postoperative ≥ 90 days, and **(C)** Overall surgery-related complication rates.

Eight studies including 1158 patients (RARC group = 619, LRC group = 539) reported the long-term complications 90 days or more after surgery, and twelve studies including 1481 patients (RARC group = 801, LRC group = 680) described the occurrence of postoperative complications (short-term or long-term). Contrary to early complications at 30 days postoperatively, the RARC group had a significantly lower rate of long-term postoperative complications compared with the LRC group, regardless of Grade ≤ II (*P* = 0.0008; OR: 0.63, 95% CI: 0.48 to 0.82), or Grade ≥ III (*P* = 0.06; OR: 0.59, 95% CI: 0.40 to 0.86), while the overall postoperative complication rate was still lower in the RARC group than in the LRC group (*P* = 0.01; OR: 0.52, 95% CI: 0.32 to 0.85), as shown in **Figure 6**. All the data of the research project are summarized in **Table 3**.

**Table 3:** Summary of research results.

The research project	-OR/MD	95% CI	P
Total operative time	17.43	[-5.06,39.91]	0.13
Amount of blood loss	-82.56	[-145.04 ,-20.08]	0.01
Transfusion rate	0.77	[0.53 ,1.13]	0.18
Hospital stay	-0.66	[-2.07 , 0.76]	0.36
Days to oral intake	-0.43	[-1.33 , 0.48]	0.35
Days to regular diet	0.17	[-0.22 , 0.55]	0.40
Mean lymph-node yield	1.40	[-0.70 , 3.50]	0.19
Positive lymph nodes	0.89	[0.57, 1.39]	0.61
Positive surgical margins	1.23	[0.69 ,2.19]	0.49
Cancer-related mortality	0.75	[0.17 , 3.38]	0.71
Intraoperative complications	0.64	[0.32, 1.30]	0.22
Grade ≤ II within 30d	0.95	[0.68 , 1.34]	0.79
Grade ≥ III within 30d	0.95	[0.60 , 1.47]	0.8
Early complications within 30d	0.85	[0.41 ,1.76]	0.66
Grade ≤ II postoperative ≥ 90d	0.63	[0.48 , 0.82]	0.0008
Grade ≥ III postoperative ≥ 90d	0.59	[0.40 , 0.86]	0.006
Overall complication rate	0.52	[0.32, 0.85]	0.01

### Sensitivity analysis and subgroup analysis

Sensitivity analysis and subgroup analysis were used to find sources of heterogeneity, and minimize the impact of heterogeneity on the stability of results. After removing one study, the heterogeneity of some indicators (transfusion rate and cancer-related death) were significantly reduced among other studies. The results of sensitivity or subgroup analysis, such as transfusion rate and total complication rate, were consistent with previous results (**Table 4**). As for cancer-related death, the results after sensitivity analysis contradicted the previous results, and the mortality was significantly lower in the RARC group than in the LRC group.

**Table 4:** The results of sensitivity or subgroup analysis.

Indicators	MD/OR	*p*	*I²*
Sensitivity analysis			
Transfusion rate	0.70 [0.48,1,01]	0.06	34%
Cancer-related death	0.37 [0.21,0.93]	0.0003	0
Subgroup analysis			
Overall complication rate			
Mean age ≥ 70	0.14 [0.04,0.52]	0.004	0
Mean age < 70	0.60 [0.36,0.98]	0.04	73%

## Discussion

In this meta-analysis, 16 studies were included to determine the difference in efficacy between RARC and LRC in bladder cancer. The results showed that, compared with LRC, RARC significantly reduced surgical blood loss and reduced the incidence of postoperative complications, especially long-term complications of 90 days or longer. This may be related to the robotic arm of the robotic surgical system being very stable, avoiding the slight jitter of the human hand, and the robotic endoscope wrist is more flexible in the space that the human hand cannot reach, which is easier to protect the nerves and blood vessels, to achieve less trauma, less bleeding and fewer postoperative complications. In addition, the excellent image processing system of robot-assisted laparoscopic surgery makes the surgical field completely reach the real 3D stereoscopic effect, while the function of magnifying 10 times makes the operation more precise, and the anatomical level of blood vessels and nerves is clearer, which is more beneficial for retaining blood vessels and nerves (21, 37, 38).

Because invasive bladder cancer is a fatal disease, adequate marginal resection and pelvic lymph node dissection are important components of surgical treatment, and the quality of lymph node dissection is a key factor for the efficacy of radical cystectomy (21). When the positive surgical margin and positive lymph nodes are less during cystectomy, the survival rate is improved. Our meta-analysis indicated that there was no significant difference between RARC and LRC in the number of lymph node removals, positive lymph nodes, and positive surgical margins. Of course, whether RARC and LRC differ in the effectiveness of tumor surgery still requires long-term follow-up to determine. Other indicators, such as total operative time, short-term recovery, length of hospital stay, bladder cancer-related mortality (based on short-term follow-up results), intraoperative complications, and early complications within 30 days after surgery, there is no significant difference between the two groups. This is in agreement with the results of a network meta-analysis of open, laparoscopic, and robotic-assisted radical cystectomy for bladder cancer performed by Feng D et al, with no significant difference in lymph node yield, positive surgical margins, operating time, transfusion rate, length of stay and days to regular diet between the two groups (39).

There is an interesting phenomenon in our meta-analysis. After we excluded the literature of Khan and M. S (24) in the sensitivity analysis, the disease-related mortality was significantly reduced (*P* < 0.05), which may be related to the small sample size or unexpected errors. In the future, we need larger randomized controlled studies to verify the potential association. And the efficacy of RARC vs LRC in the treatment of bladder cancer is shown in **Table 5**.

**Table 5:** The summary of the efficacy of RARC vs LRC in the treatment of bladder cancer.

RARC versus LRC in Bladder cancer																
Intraoperative Security			Postoperative recovery			Oncologic outcomes				Complications						
Total operative time	Amount of blood loss	Transfusion rate	Hospital stay	Day to oral intake	Day to regular diet	Mean lymph node yield	Positive lymph node	Positive surgical margins	Cancer-related mortality	Intraoperative complication	Postoperative within 30days complication Grade ≤ II	Postoperative within 30days complication Grade ≥ III	Early complications within 30d	Postoperative 90 days or longer complication Grade ≤ II	Postoperative 90 days or longer complication Grade ≥ III	Overall surgery-related complication
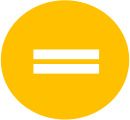	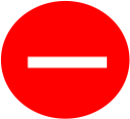	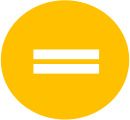	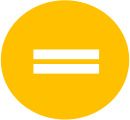	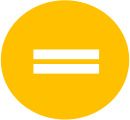	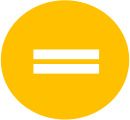	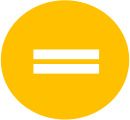	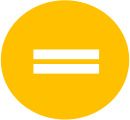	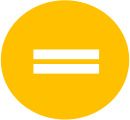	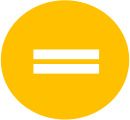	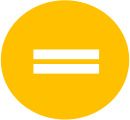	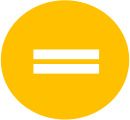	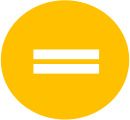	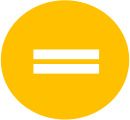	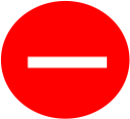	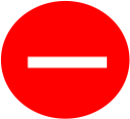	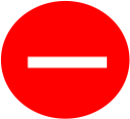

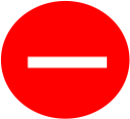
 means RARC decreased the effect vs LRC group; 
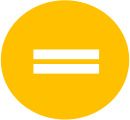
 means that RARC has the same effect vs LRC group.

RARC, robotic assisted radical cystectomy; LRC, laparoscopic radical cystectomy.

In addition, in order to evaluate the cost-effectiveness, we simply analyzed the cost of RARC and LRC, although the surgical outcome is crucial, after all, medical cost is also one of the main concerns of patients. Based on current evidence, RARC has a lower complication rate compared with LRC, but it is more costly. Based on the findings of Morii Y et al. (40), the operating cost of robotic surgery accounted for 63.1-70.5% of the total surgical cost. Interestingly, robotics-related costs accounted for a lower proportion of total surgical costs in institutions with more cases, and conversely, robotics-related costs accounted for a larger proportion of total surgical costs. Therefore, the most effective way to reduce the costs associated with robotic surgery is to shorten the operation time and increase the number of cases. But in addition to focusing on the cost of surgery, when studying the cost-effectiveness of surgery, quality measures such as quality of life and survival, and even costs related to the treatment of complications need to be considered. But there are also some European countries where RARC costs depend mainly on patient hospital stay and surgery time, rather than robotic instruments (41). Unfortunately, the studies we included did not report the cost-effectiveness-related indicators of the two types of surgery, and we were unable to draw conclusions by integrating them. In addition, the port site metastatic rate and intrabdominal seeding rate, which we were concerned about, were not mentioned in our included studies. And, these are the hotspots that need to be paid attention to in future RARC and LRC-related research.

In this meta-analysis, we created a more precise classification of complications to compare the differences between the two groups, in order to more comprehensively and accurately evaluate the prognosis of the two surgical patients and provide more information for clinicians, which was not available in prior meta-analyses. In addition, new evidence on the efficacy and safety of RARC and LRC has been published in recent years, and some of the results are controversial. Our meta-analysis combined recent studies to clarify their pros and cons in bladder cancer treatment.

There are some limitations of this meta-analysis that should be noted: (1) there were differences in the inclusion and exclusion criteria for patients among the included studies; (2) the surgeons are different among the studies; (3) only studies that were published in English and Chinese were considered for inclusion, thus we may have missed some studies that satisfied the inclusion criteria; (4) there is large heterogeneity in partial analysis results that affects the stability of the results, and we tried to conduct sensitivity analysis and subgroup analysis but still cannot fully identify the source of heterogeneity.

In conclusion, our results suggest that RARC is a safe and effective treatment for bladder cancer patients as LRC. Patients with RARC might benefit from significantly less blood loss and fewer postoperative complications, especially long-term complications 90 days or more after surgery. However, despite the methodological review, due to the limitations associated with the included studies and our analysis, additional large sample-size, prospective, multi-center, long-term follow-up studies, and randomized control trials are needed to confirm this conclusion.

## Data availability statement

The original contributions presented in the study are included in the article/**Supplementary Material**. Further inquiries can be directed to the corresponding authors.

## Author contributions

JL and LW wrote the main manuscript text, ND, XB, and SC prepared tables and figures, SS revised and polished the manuscript, HL and YL proposed research themes and coordinated work within the group. All authors reviewed the manuscript and agreed on the journal to which the article should be submitted.

## Funding

This work was supported by the Wu Jieping Medical Foundation (No. 320.6750.2020-21-12). The sponsor will not be involved in study design, collection, management, analysis, and interpretation of data, writing of the report, and the decision to submit the report for publication.

## Conflict of interest

The authors declare that the research was conducted in the absence of any commercial or financial relationships that could be construed as a potential conflict of interest.

## Publisher’s note

All claims expressed in this article are solely those of the authors and do not necessarily represent those of their affiliated organizations, or those of the publisher, the editors and the reviewers. Any product that may be evaluated in this article, or claim that may be made by its manufacturer, is not guaranteed or endorsed by the publisher.

## References

[1] Sung H Ferlay J Siegel RL Laversanne M Soerjomataram I Jemal A . Global cancer statistics 2020: GLOBOCAN estimates of incidence and mortality worldwide for 36 cancers in 185 countries. CA Cancer J Clin (2021) 71(3):209–49. doi: 10.3322/caac.21660 33538338

[2] Wong MCS Fung FDH Leung C Cheung WWL Goggins WB Ng CF . The global epidemiology of bladder cancer: A joinpoint regression analysis of its incidence and mortality trends and projection. Sci Rep (2018) 8(1):1129. doi: 10.1038/s41598-018-19199-z 29348548 PMC5773684

[3] Kiss B Burkhard FC Thalmann GN . Open radical cystectomy: still the gold standard for muscle invasive bladder cancer. World J Urol (2016) 34(1):33–9. doi: 10.1007/s00345-015-1729-7 26586476

[4] Dinney CP . Therapy of invasive bladder cancer. Urology (2006) 67(3 Suppl 1):56–61. doi: 10.1016/j.urology.2006.01.043 16530078

[5] Stein JP Lieskovsky G Cote R Groshen S Feng AC Boyd S . Radical cystectomy in the treatment of invasive bladder cancer: long-term results in 1,054 patients. J Clin Oncol (2001) 19(3):666–75. doi: 10.1200/JCO.2001.19.3.666 11157016

[6] Parra RO Andrus CH Jones JP Boullier JA . Laparoscopic cystectomy: initial report on a new treatment for the retained bladder. J Urol (1992) 148(4):1140–4. doi: 10.1016/s0022-5347(17)36843-x 1404624

[7] Sánchez de Badajoz E Gallego Perales JL Reche Rosado A Gutierrez de la Cruz JM Jimenez Garrido A . Laparoscopic cystectomy and ileal conduit: case report. J Endourol (1995) 9(1):59–62. doi: 10.1089/end.1995.9.59 7780433

[8] Wang SZ Chen LW Zhang YH Wang WW Chen W Lin HY . Comparison of hand-assisted laparoscopic and open radical cystectomy for bladder cancer. Urol Int (2010) 84(1):28–33. doi: 10.1159/000273462 20173365

[9] Menon M Hemal AK Tewari A Shrivastava A Shoma AM El-Tabey NA . Nerve-sparing robot-assisted radical cystoprostatectomy and urinary diversion. BJU Int (2003) 92(3):232–6. doi: 10.1046/j.1464-410x.2003.04329.x 12887473

[10] Maes AA Brunkhorst LW Gavin PW Todd SP Maatman TJ . Comparison of robotic-assisted and open radical cystectomy in a community-based, non-tertiary health care setting. J Robot Surg (2013) 7(4):359–63. doi: 10.1007/s11701-013-0401-8 27001875

[11] Nguyen DP Al Hussein Al Awamlh B Charles Osterberg E Chrystal J Flynn T Lee DJ . Postoperative complications and short-term oncological outcomes of patients aged ≥80 years undergoing robot-assisted radical cystectomy. World J Urol (2015) 33(9):1315–21. doi: 10.1007/s00345-014-1446-7 25410374

[12] Richards KA Kader AK Otto R Pettus JA Smith JJ 3rd Hemal AK . Is robot-assisted radical cystectomy justified in the elderly? a comparison of robotic versus open radical cystectomy for bladder cancer in elderly ≥75 years old. J Endourol (2012) 26(10):1301–6. doi: 10.1089/end.2012.0035 22582706

[13] Son SK Lee NR Kang SH Lee SH . Safety and effectiveness of robot-assisted versus open radical cystectomy for bladder cancer: A systematic review and meta-analysis. J Laparoendosc Adv Surg Tech A (2017) 27(11):1109–20. doi: 10.1089/lap.2016.0437 28350238

[14] Catto JWF Khetrapal P Ricciardi F Ambler G Williams NR Al-Hammouri T . Effect of robot-assisted radical cystectomy with intracorporeal urinary diversion vs open radical cystectomy on 90-day morbidity and mortality among patients with bladder cancer: A randomized clinical trial. JAMA (2022) 327(21):2092–103. doi: 10.1001/jama.2022.7393 PMC9109000 35569079

[15] Li K Lin T Fan X Xu K Bi L Duan Y . Systematic review and meta-analysis of comparative studies reporting early outcomes after robot-assisted radical cystectomy versus open radical cystectomy. Cancer Treat Rev (2013) 39(6):551–60. doi: 10.1016/j.ctrv.2012.11.007 23273846

[16] Tang K Li H Xia D Hu Z Zhuang Q Liu J . Laparoscopic versus open radical cystectomy in bladder cancer: a systematic review and meta-analysis of comparative studies. PloS One (2014) 9(5):e95667. doi: 10.1371/journal.pone.0095667 24835573 PMC4023936

[17] Jadad AR Moore RA Carroll D Jenkinson C Reynolds DJ Gavaghan DJ . Assessing the quality of reports of randomized clinical trials: is blinding necessary? Control Clin Trials (1996) 17(1):1–12. doi: 10.1016/0197-2456(95)00134-4 8721797

[18] Grisar K Chaabouni D Romero LPG Vandendriessche T Politis C Jacobs R . Autogenous transalveolar transplantation of maxillary canines: A systematic review and meta-analysis. Eur J Orthod (2018) 40(6):608–16. doi: 10.1093/ejo/cjy026 PMC6265659 29860316

[19] Zhong Y Huo H Dai S Li S . Efficacy and safety of immune checkpoint inhibitors-combined antiangiogenic drugs in the treatment of hepatocellular carcinoma: A systematic review and meta analysis. Front Oncol (2022) 12:964779. doi: 10.3389/fonc.2022.964779 36059696 PMC9433548

[20] Teishima J Hieda K Inoue S Goto K Ikeda K Ohara S . Comparison of initial experiences of robot-assisted radical cystectomy with those of laparoscopic for bladder cancer. Innov (Phila) (2014) 9(4):322–6. doi: 10.1097/IMI.0000000000000056 25062101

[21] Abraham JB Young JL Box GN Lee HJ Deane LA Ornstein DK . Comparative analysis of laparoscopic and robot-assisted radical cystectomy with ileal conduit urinary diversion. J Endourol (2007) 21(12):1473–80. doi: 10.1089/end.2007.0095 18186686

[22] Gastecka A Hnatyszyn-Dzikowska A Hejka P Adamczyk P Pokryw zynska M Kloskowski T . Cost comparison of laparoscopic versus robot-assisted radical cystectomy. Health Policy Technol (2018) 7:420–6. doi: 10.1016/j.hlpt.2018.10.008

[23] Jia GZ Liu A Dong K Zhang Z Hou J Gao X . Comparative study on curative effect and complication rate among open, laparoscopic and robotic radical cystectomy: report of 325 cases. J Clin Urol (Chinese) (2017) 32(01):42–5. doi: 10.13201/j.issn.1001-1420.2017.01.011

[24] Khan MS Challacombe B Elhage O Rimington Coker B Murphy D . A dual-centre, cohort comparison of open, laparoscopic and robotic-assisted radical cystectomy. Int J Clin Pract (2012) 66(7):656–62. doi: 10.1111/j.1742-1241.2011.02888.x 22507234

[25] Khan MS Gan C Ahmed K Watkins J Summers JA Peacock JL . A single-centre early phase randomised controlled three-arm trial of open, robotic, and laparoscopic radical cystectomy (CORAL). Eur Urol (2016) 69(4):613–21. doi: 10.1016/j.eururo.2015.07.038 26272237

[26] Kim TH Sung HH Jeon HG Seo SI Jeon SS Lee HM . Oncological outcomes in patients treated with radical cystectomy for bladder cancer: Comparison between open, laparoscopic, and robot-assisted approaches. J Endourol (2016) 30(7):783–91. doi: 10.1089/end.2015.0652 27055782

[27] Matsumoto K Tabata KI Hirayama T Shimura S Nishi M Ishii D . Robot-assisted laparoscopic radical cystectomy is a safe and effective procedure for patients with bladder cancer compared to laparoscopic and open surgery: Perioperative outcomes of a single-center experience. Asian J Surg (2019) 42(1):189–96. doi: 10.1016/j.asjsur.2017.11.002 29254869

[28] Wei XS Zhuang QY Hu Z Liu Z Wang Z Li F . Clinical analysis of robot-assisted laparoscopic, traditional laparoscopic and open radical cystectomy with bricker ideal neobladder. J Contemp Urol Reprod Oncol (Chinese) (2016) 8(02):76–81. doi: 10.3870/j.issn.1674-4624.24.2016.02.004

[29] Bai Y Wang S Zheng W Li E Quan J Wei F . Clinical outcome of laparoscopic versus robot-assisted radical cystectomy for patients with bladder cancer: a retrospective study. BMC Surg (2021) 21(1):388. doi: 10.1186/s12893-021-01382-1 34727908 PMC8561927

[30] Arora A Pugliesi F Zugail AS Moschini M Pazeto C Macek P . Comparing perioperative complications between laparoscopic and robotic radical cystectomy for bladder cancer. J Endourol (2020) 34(10):1033–40. doi: 10.1089/end.2020.0112 32597214

[31] Su S Gu L Ma X Li H Wang B Shi T . Comparison of laparoscopic and robot-assisted radical cystectomy for bladder cancer: Perioperative and oncologic outcomes. Clin Genitourin Cancer (2019) 17(5):e1048–53. doi: 10.1016/j.clgc.2019.06.007 31303560

[32] Zhang S Lin T Zhang Q Zhang S Liu G Ji C . Comparison of perioperative outcomes in robot-assisted radical cystectomy and laparoscopic radical cystectomy. Int J Med Robot (2020) 16(2):e2074. doi: 10.1002/rcs.2074 31922333

[33] Porreca A Di Gianfrancesco L Artibani W Busetto GM Carrieri G Antonelli A . Robotic-assisted, laparoscopic, and open radical cystectomy: Surgical data of 1400 patients from the Italian radical cystectomy registry on intraoperative outcomes. Cent Eur J Urol (2022) 75(2):135–44. doi: 10.5173/ceju.2022.0284 PMC9326698 35937656

[34] Jiang S Xu P Xiang Z Wang H Sun L Guo J . Comparison of perioperative outcomes of robot-assisted laparoscopic, traditional laparoscopic and open radical cystectomy with ileal conduit. Fudan Univ J Med Sci (Chinese) (2020) 47(1):1–6. doi: 10.3969/j.issn.1672-8467.2020.01.001

[35] Huang X Zheng W Wang S Qi X Zhang Q Liu F . Comparison of robot-assisted and laparoscopic radical cystectomy. Zhejiang med(Chinese) (2019) 41(07):694–6. doi: 10.12056/j.issn.1006-2785.2019.41.7.2018-1724

[36] Dindo D Demartines N Clavien PA . Classification of surgical complications: a new proposal with evaluation in a cohort of 6336 patients and results of a survey. Ann Surg (2004) 240(2):205–13. doi: 10.1097/01.sla.0000133083.54934.ae PMC1360123 15273542

[37] Nix J Smith A Kurpad R Nielsen ME Wallen EM Pruthi RS . Prospective randomized controlled trial of robotic versus open radical cystectomy for bladder cancer: perioperative and pathologic results. Eur Urol (2010) 57(2):196–201. doi: 10.1016/j.eururo.2009.10.024 19853987

[38] Snow-Lisy DC Campbell SC Gill IS Hernandez AV Fergany A Kaouk J . Robotic and laparoscopic radical cystectomy for bladder cancer: Long-term oncologic outcomes. Eur Urol (2014) 65(1):193–200. doi: 10.1016/j.eururo.2013.08.021 24018019

[39] Feng D Li A Hu X Lin T Tang Y Han P . Comparative effectiveness of open, laparoscopic and robot-assisted radical cystectomy for bladder cancer: a systematic review and network meta-analysis. Minerva Urol Nefrol (2020) 72(3):251–64. doi: 10.23736/S0393-2249.20.03680-2 32083418

[40] Morii Y Osawa T Suzuki T Shinohara N Harabayashi T Ishikawa T . Cost comparison between open radical cystectomy, laparoscopic radical cystectomy, and robot-assisted radical cystectomy for patients with bladder cancer: A systematic review of segmental costs. BMC Urol (2019) 19(1):110. doi: 10.1186/s12894-019-0533-x 31703573 PMC6842244

[41] Mjaess G Diamand R Aoun F Assenmacher G Assenmacher C Verhoest G . Cost-analysis of robot-assisted radical cystectomy in Europe: A cross-countrycomparison. Eur J Surg Oncol: J Euro Soc Surg Oncol Brit Assoc Surg Oncol (2022) S0748-7983(22):00584–4. doi: 10.1016/j.ejso.2022.07.023 35970622

