# Supplementary material for: Robotic-assisted versus standard laparoscopic radical cystectomy in bladder cancer: A systematic review and meta-analysis
Source: Front Oncol. 2022 Nov 9;12:1024739. doi: 10.3389/fonc.2022.1024739 (PMC9681903; doi:10.3389/fonc.2022.1024739)
Supplement: Supplementary file 1 [file DataSheet_1.docx]

Date Run: 29/7/2022 12:27

**Search strategy of PUBMED**

ID Search Hits

#1 Search "Cystectomy"[Mesh] 10367

#2 Search Cystectomies[Title/Abstract] 639

#3 #1 or #2 10699

#4 Search "Laparoscopy"[Mesh] 112155

#5 Search: **((((((((((((((((((Laparoscopies[Title/Abstract]) OR (Celioscopy[Title/Abstract])) OR (Celioscopies[Title/Abstract])) OR (Peritoneoscopy[Title/Abstract])) OR (Peritoneoscopies[Title/Abstract])) OR (Surgical Procedures, Laparoscopic[Title/Abstract])) OR (Laparoscopic Surgical Procedure[Title/Abstract])) OR (Procedure, Laparoscopic Surgical[Title/Abstract])) OR (Procedures, Laparoscopic Surgical[Title/Abstract])) OR (Surgery, Laparoscopic[Title/Abstract])) OR (Laparoscopic Surgical Procedures[Title/Abstract])) OR (Laparoscopic Surgery[Title/Abstract])) OR (Laparoscopic Surgeries[Title/Abstract])) OR (Surgeries, Laparoscopic[Title/Abstract])) OR (Laparoscopic Assisted Surgery[Title/Abstract])) OR (Laparoscopic Assisted Surgeries[Title/Abstract])) OR (Surgeries, Laparoscopic Assisted[Title/Abstract])) OR (Surgery, Laparoscopic Assisted[Title/Abstract])) OR (Surgical Procedure, Laparoscopic[Title/Abstract]) 27383**

#6 #4 or #5 120173

#7 Search "Robotic Surgical Procedures"[Mesh] 13641

#8 Search: **(((((((((((((((((((((((Procedure, Robotic Surgical[Title/Abstract]) OR (Procedures, Robotic Surgical[Title/Abstract])) OR (Robotic Surgical Procedure[Title/Abstract])) OR (Surgical Procedure, Robotic[Title/Abstract])) OR (Robot Surgery[Title/Abstract])) OR (Robot Surgeries[Title/Abstract])) OR (Surgery, Robot[Title/Abstract])) OR (Robot-Assisted Surgery[Title/Abstract])) OR (Robot Assisted Surgery[Title/Abstract])) OR (Robot-Assisted Surgeries[Title/Abstract])) OR (Surgery, Robot-Assisted[Title/Abstract])) OR (Robot-Enhanced Procedures[Title/Abstract])) OR (Procedure, Robot-Enhanced[Title/Abstract])) OR (Robot Enhanced Procedures[Title/Abstract])) OR (Robot-Enhanced Procedure[Title/Abstract])) OR (Surgical Procedures, Robotic[Title/Abstract])) OR (Robotic-Assisted Surgery[Title/Abstract])) OR (Robotic Assisted Surgery[Title/Abstract])) OR (Robotic-Assisted Surgeries[Title/Abstract])) OR (Surgery, Robotic-Assisted[Title/Abstract])) OR (Robot-Enhanced Surgery[Title/Abstract])) OR (Robot Enhanced Surgery[Title/Abstract])) OR (Robot-Enhanced Surgeries[Title/Abstract])) OR (Surgery, Robot-Enhanced[Title/Abstract]) 2665**

#9 #7 or #8 15103

#10 #3 and #6 and #9 91

#11 Search randomized controlled trial[Publication Type] OR randomized[Title/Abstract] OR placebo[Title/Abstract] 743763

#12 #10 and #11 10

**Search strategy of EMBASE**

ID Search Hits

#1 'Cystectomy'/exp 33961

#2 'bladder extirpation':ab,ti OR 'bladder resection':ab,ti OR 'bladder resection, partial':ab,ti OR 'cystectomy, partial':ab,ti OR 'cystectomy, total':ab,ti OR 'cystoprostatectomy':ab,ti OR 'partial bladder resection':ab,ti OR 'partial cystectomy':ab,ti OR 'pericystectomy':ab,ti OR 'radical cystectomy':ab,ti OR 'total cystectomy':ab,ti OR 'urinary bladder resection':ab,ti 20057

#3 #1 OR #2 36738

#4 'Laparoscopy'/exp 185537

#5 'laparoscopy, video':ab,ti OR 'pelvic endoscopy':ab,ti OR 'peritoneoscopy':ab,ti OR 'videolaparoscopy':ab,ti 1194

#6 #4 or #5 185751

#7 'Robot assisted surgery'/exp 24915

#8 'robot aided surgery':ab,ti OR 'robot surgery':ab,ti OR 'robotic aided surgery':ab,ti OR 'robotic surgery':ab,ti OR 'robotic surgical procedure':ab,ti OR 'robotic surgical procedures':ab,ti OR 'robotically assisted surgery':ab,ti 10629

#9 #7 OR #8  29499

#10 #3 AND #6 AND #9 453

#11 'clinical trial'/exp 1732007

#12 'clinical':ab,ti AND 'trial':ab,ti OR  ’random’ OR 'drug therapy' 5930497

#13 #11 OR #12 6662353

#14 #3 AND #6 AND #9 AND #14 83

**Search strategy of cochrane**

Search Name:

Date Run: 30/07/2022 06:32:06

Comment:

ID Search Hits

#1 MeSH descriptor: [Cystectomy] explode all trees 305

#2 (cystectomies):ti,ab,kw (Word variations have been searched) 1765

#3 #1 or #2 1765

#4 MeSH descriptor: [Laparoscopy] explode all trees 6492

#5 (Procedures, Laparoscopic Surgical or Surgery, Laparoscopic Assisted or Laparoscopic Surgeries or Laparoscopic Surgical Procedure or Procedure, Laparoscopic Surgical):ti,ab,kw (Word variations have been searched) 16832

#6 (Surgeries, Laparoscopic or Laparoscopic Assisted Surgeries or Laparoscopic Surgical Procedures or Laparoscopic Assisted Surgery or Surgery, Laparoscopic):ti,ab,kw (Word variations have been searched) 16833

#7 (Surgical Procedures, Laparoscopic or Surgical Procedure, Laparoscopic or Surgeries, Laparoscopic Assisted or Laparoscopic Surgery; Celioscopy or Peritoneoscopy; Laparoscopies):ti,ab,kw (Word variations have been searched) 6101

#8 (Celioscopies or Peritoneoscopies):ti,ab,kw (Word variations have been searched) 30

#9 #4 or #5 or #6 or #7 or #8 18192

#10 MeSH descriptor: [Robotic Surgical Procedures] explode all trees 412

#11 (Robot-Enhanced Surgeries or Robot-Enhanced Surgery or Surgery, Robot-Enhanced or Robot Enhanced Surgery or Robot Surgery or Procedure, Robot-Enhanced):ti,ab,kw (Word variations have been searched) 2853

#12 (Surgery, Robotic-Assisted or Surgical Procedures, Robotic or Surgery, Robot or Robot Enhanced Procedures or Robot-Assisted Surgery):ti,ab,kw (Word variations have been searched) 4242

#13 (Surgery, Robotic-Assisted or Surgical Procedures, Robotic or Surgery, Robot or Robot Enhanced Procedures or Robot-Assisted SurgeryRobotic-Assisted Surgeries or Robotic Assisted Surgery or Surgical Procedure, Robotic or Robot Assisted Surgery or Robot-Enhanced Proceduresor Robot-Enhanced Procedure):ti,ab,kw (Word variations have been searched) 4249

#14 (Procedures, Robotic Surgical or Robot Surgeries or Robot-Assisted Surgeries or Procedure, Robotic Surgical or Robotic-Assisted Surgery or Surgery, Robot-Assisted or Robotic Surgical Procedure):ti,ab,kw (Word variations have been searched) 4219

#15 #10 or #11 or #12 or #13 or #14 4249

#16 #3 and #9 and #15 65

**Search strategy of CBM**

ID Search Hits

#1 "膀胱切除术"[不加权:扩展] 13469

#2 膀胱切除术 21574

#3 (#1) OR (#2) 21574

#4 "腹腔镜检查"[不加权:扩展] 209828

#5 腹腔镜检术 or 腹腔镜检 or 外科, 腹腔镜 or 腹腔镜外科手术 273324

#6 (#4) OR (#5) 273324

#7 "机器人"[不加权:扩展] 30680

#8 机器人外科手术 102

#9 (#7) OR (#8) 30685

#10 (#3) AND (#6) AND (#9) 238

#11 "随机对照试验"[不加权:扩展] 504599

#12 随机对照试验 723798

#13 (#9) OR (#8) 723798

#14 (#3) AND (#6) AND (#9) AND (#12) 4
